# Reduced Transmissibility of East African Indian Strains of *Mycobacterium tuberculosis*

**DOI:** 10.1371/journal.pone.0025075

**Published:** 2011-09-19

**Authors:** Amr S. Albanna, Michael B. Reed, Kimberley V. Kotar, Ashley Fallow, Fiona A. McIntosh, Marcel A. Behr, Dick Menzies

**Affiliations:** 1 Respiratory Epidemiology and Clinical Research Unit, Montreal Chest Institute, McGill University, Montreal, Quebec, Canada; 2 Research Institute of the McGill University Health Centre, Montreal General Hospital, Montreal, Quebec, Canada; Institut de Pharmacologie et de Biologie Structurale, France

## Abstract

**Background:**

*Mycobacterium tuberculosis* (MTB) has been classified into 4 main lineages. Some reports have associated certain lineages with particular clinical phenotypes, but there is still insufficient information regarding the clinical and epidemiologic implications of MTB lineage variation.

**Methods:**

Using large sequence polymorphisms we classified MTB isolates from a population-based study in Montreal, Canada into the 4 major lineages, and identified the associated clinical and epidemiologic features. In addition, IS6110-RFLP and spoligotyping were used as indicators of recent TB transmission. The study population was divided into a derivation cohort, diagnosed between 2001 and 2007, and a separate validation cohort, diagnosed between 1996 and 2000.

**Results:**

In the derivation cohort, when compared to the other MTB lineages, the East African-Indian (EAI) lineage was associated with lower rates of TB transmission, as measured by: positive TST among close contacts of pulmonary TB cases (adjusted odds ratio 0.6: [95% confidence interval 0.4–0.9]), and clustered TB cases (0.3: [<0.001–0.6]). Severe forms of TB were also less likely among the EAI group (0.4: [<0.001–0.8]). There were no significant differences when comparing patients with the other MTB lineages. In the validation cohort, the EAI lineage was associated with lower rates of positive TST among contacts (0.5: [0.3–0.9]) and a trend towards less clustered TB cases (0.5: [0.1–1.8]) when compared to the other lineages. Disease severity among the different groups was not significantly different in the validation cohort.

**Conclusions:**

We conclude that in Montreal, EAI strains were associated with reduced transmission compared to other MTB lineages.

## Introduction

Tuberculosis (TB) is a contagious disease caused by *Mycobacterium tuberculosis* (MTB). Despite the availability of adequate diagnostic tests and effective treatment, TB accounts for 9.2 million new illnesses and nearly 2 million deaths annually [1]. This is explained in part by environmental risk factors, such as poorly ventilated and crowded conditions, poverty, and lack of access to medical care. Host-related factors, such as malnutrition or HIV co-infection, play a major role, but the contribution of bacterial factors is relatively unknown. Recently a robust method has been developed to define major MTB lineages, which is based on the detection of large sequence polymorphisms (LSPs) or single nucleotide polymorphisms (SNPs) [2]–[4]. Studies using these techniques have identified six major lineages, 4 sub-divide MTB and 2 sub-divide *M. africanum* [3], [5]. These lineages have distinct geographic distributions and have been tentatively linked with differing epidemiologic and clinical profiles.

Experimental laboratory studies have provided evidence suggesting that distinct MTB strains may have variable virulence properties. For example, there are several reports describing apparently enhanced *in vivo* virulence of certain members of the “Beijing” lineage [6]–[8]. In at least some instances, this enhanced virulence appears to be related to the production of a complex phenolic glycolipid (PGL-tb) which inhibits macrophage proinflammatory cytokine release [9], [10]. However there is little published evidence that these laboratory findings are associated with important clinical consequences. Hence environmental and host related risk factors are still considered the most important factors influencing disease transmission and severity.

Combined data from 49 studies concluded that the Beijing lineage has emerged in many areas of the world including Cuba, the former Soviet Union, Vietnam, South Africa, Malawi, and Argentina [11]. For example, Beijing strains did not exist in Cape Town (South Africa) prior to 1965, but now account for 20% of all TB cases there. Interestingly, in these Cape Town studies, patients infected with the Beijing genotype were more likely to be heavily AFB smear positive in their sputum [12]–[14]. These findings have led to the suggestion that strains belonging to the Beijing lineage possess unique attributes that confer an increased ability to cause disease and to transmit within certain geographic settings or ethnic groups [15]. In contrast, in a prospective study of the development of active TB among close contacts of patients with active TB, the proportion with disease caused by *M. africanum* was significantly lower than with other MTB strains [16]. Very little information is presently available regarding clinical features attributable to the other main TB lineages, including the East African-Indian (EAI) lineage that is the focus of the current study.

The objective of the present study was to investigate the association of the major MTB lineages with: transmissibility of infection and disease, disease severity, and drug resistance to first line anti-TB drugs.

## Methods

### Study population

The study cohort consisted of all persons resident in Montreal diagnosed with active TB between January 1996 and May 2007. Clinical and demographic data for the TB cases, and their close contacts was obtained from the records of the Public Health Unit, and hospital medical records.

This cohort of TB cases was divided into a derivation cohort, diagnosed between January 2001 and May 2007, and a validation cohort, diagnosed between January 1996 and December 2000. Because the analyses in the derivation cohort identified the EAI lineage as potentially of greatest interest, we restricted the validation cohort to persons born in countries (Afghanistan, Bangladesh, Djibouti, Ethiopia, India, Kenya, Pakistan, Somalia, Sri Lanka, and Tanzania) where EAI strains are commonly encountered [3], [5].

We included TB patients, whose TB diagnosis was based on culture isolation of MTB, and household or family contacts of pulmonary TB patients. HIV co-infected patients and their contacts and patients infected with *M. Africanum* and their contacts were excluded.

### Ethics statement

The study was approved by the Biomedical - C Research ethics board of the McGill University Health Centre. Patients' consent was not required because there was no direct contact with the patients and the data was gathered anonymously. The above named research ethics board waived the need for consent.

### Laboratory methods

Mycobacterial isolates of all cohort members were retrieved from the Quebec provincial reference lab [3]. The main MTB lineages were identified using PCR-based detection of Large Sequence Polymorphisms (LSPs) as previously described [3]. The identified LSP deletions were as follows: RD105 for the East Asian or Beijing lineage, RD239 for the Indo-Oceanic lineage, RD750 for the EAI lineage, and the Euro-American lineage strains were identified using sequence analysis of a portion of the polyketide synthase 1–15 gene (*pks1-15*).

Disease transmission was assessed using IS*6110* RFLP (restriction fragment length polymorphism) [17]. Spoligotyping was performed on strains with less than 6 RFLP “bands” [3].

### Indicators of transmission and disease severity

We estimated the likelihood of a previous positive TST in each contact using the formula: *PTBI = [1−(1-ARI)^age^]* [18], where *PTBI = the probability of TB infection, ARI = the average estimated annual risk of TB infection in each contact's country of origin*, and *Age = their age when they left that country*. ARI was estimated from the WHO estimated incidence of smear positive TB in their country of origin using the Styblo formula [1], [19]. The probability of previous TB infection in each contact was summed to give an estimated proportion of previous TB infection for each lineage group, and then subtracted from the observed proportion with positive TST to estimate the proportion with recent TB infection (i.e. equivalent to TST conversion). The second transmission indicator was the proportion of active cases for each lineage group in clusters with identical RFLP or spoligotype patterns.

Severe disease was defined as: disseminated TB, TB meningitis, bilateral lung consolidation with 4 or 5 lobes involved, or death before or during anti-tuberculous therapy.

### Statistical analysis

Associations between clinical characteristics and MTB strain lineage were tested for significance using the chi-square or Fisher's exact tests for categorical, and *t* tests or analysis of variance for continuous variables. Differences in outcomes between groups were expressed as odds ratios and 95% confidence intervals. Multiple logistic regression analysis was performed to adjust for potential confounding covariates. All analyses were conducted using SAS (version 9.2) software.

## Results

### Derivation cohort

A total of 816 patients were diagnosed with culture positive active TB on the island of Montreal between January 2001 and May 2007. Of these patients 17% were born in Canada, 19.3% were foreign-born from the Americas or the Caribbean, 8.6% originated from Europe, 21.8% from Africa and the Middle East, 12.5% from the Indian subcontinent, and 20.7% from Asia. From the total number, 78 patients were excluded because of unsuccessful DNA extraction or insufficient clinical data. In addition, 62 HIV sero-positive patients were excluded because of the profound effect HIV co-infection can have on clinical outcomes [20], [21]. We also excluded patients infected with *M. africanum* due to the small numbers of patients involved (3 patients). This left 678 patients with active TB who were analyzed, of whom 466 had pulmonary disease – these patients had 1339 close contacts; for more details about population selection see Figure S1.

The most commonly isolated MTB lineage in this cohort belonged to the Euro-American lineage (66.7%), 9.6% were Beijing, 17.7% were Indo-Oceanic and 6% were EAI strains. Except for the TB incidence in the patients' countries of origin and the age of contacts, the environmental and host-related risk factors were comparable between the groups infected with EAI and non-EAI MTB lineages (Table 1).

**Table 1:** Patient characteristics associated with East African-Indian and other *Mycobacterium tuberculosis* lineages.

	Derivation Cohort			Validation cohort		
	EAI	Non-EAI	P-value	EAI	Non-EAI	P-value
**A) TB patients (i.e. index cases).**						
**Total number**	41	637		36	55	
**Age, mean (SD)**	47 (21)	44 (20)	0.38	40 (20)	36 (15)	0.3
**Male sex, N (%)**	20 (49)	337 (54)	0.53	25 (69)	37 (68.5)	0.9
**Immigrants from high TB prevalence country** * **, N (%)**	36 (88)	376 (61)	-†	36 (100)	42 (76)	-†
**Years in Canada** ‡ **, mean (SD)**	8 (10)	10 (12)	0.24	5.3 (8.2)	4.7 (5.8)	0.7
**Co-morbidities** § **, N (%)**	13 (32)	215 (35)	0.8	9 (26)	16 (31)	0.6
**History of smoking, N (%)**	5 (19)	155 (31)	0.18	6 (23)	14(33)	0.4
**Alcohol abuse, N (%)**	4(15)	89 (18)	0.69	3(12)	5 (13)	1.0
**IV drug use, N**	0	4 (0.8)	0.63	0	0	-
**Past TB infection, N (%)**	2 (5)	69 (11)	0.23	2 (6)	5 (11)	0.7
**B) Close contacts of pulmonary TB patients.**						
**Total number**	74	1265		86	143	
**Age, mean (SD)**	35 (23)	29 (20)	0.05	26 (19)	21 (16)	0.1
**Male sex** ll **, N (%)**	13 (45)	170 (51)	0.55	-	-	-
**Immigrants from high TB prevalence country** * **, N (%)**	46 (62)	675 (53)	-†	84 (98)	121 (85)	-†
**Years in Canada** ‡ **, mean (SD)**	10 (10)	9 (10)	0.7	5.3 (8)	3.6 (3.8)	0.08

Abbreviations: EAI = East African-Indian; TB = tuberculosis; N = number; SD = standard deviation IV = intravenous.

*Estimated TB prevalence >150 per 100, 000, based on the 2009 World Health Organization reports (1);

† country of origin was basis for assessment into groups, hence statistical testing would not be appropriate;

‡ number of years of stay in Canada since immigration – for foreign born only;

§ co-morbidities include chronic pulmonary diseases, diabetes, cardiac diseases, renal diseases, liver diseases, and non-HIV immunosuppressive disorders (e.g. malignancies, immunosuppressive medications);

ll 73% missing values in the derivation cohort and completely missing in the validation cohort.

For comparison between the 3 major non-EAI lineages see Table S1.

**Figure 1:**
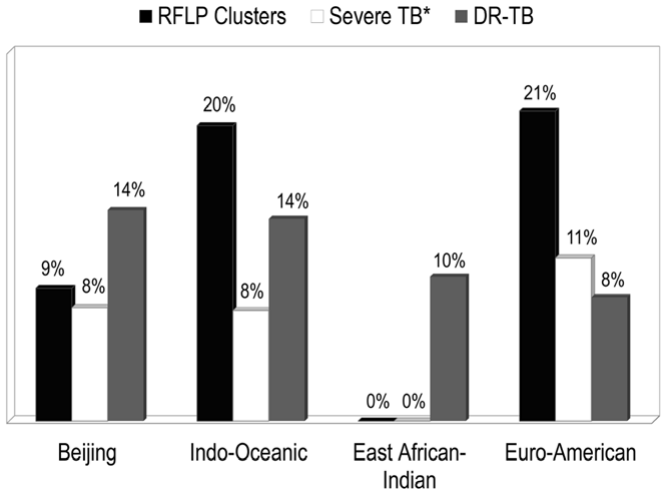
Proportion of recent tuberculosis transmission, severe disease, and drug resistant tuberculosis among derivation cohort patients. A comparison between patients within each MTB lineage group. Recent TB transmission is indicated by RFLP clusters. Abbreviations: RFLP = restriction fragment length polymorphism; DR-TB = drug resistant tuberculosis. *Severe TB indicates disseminated TB, TB meningitis, extensive bilateral lung consolidation or death during the course of TB therapy.

**Figure 2:**
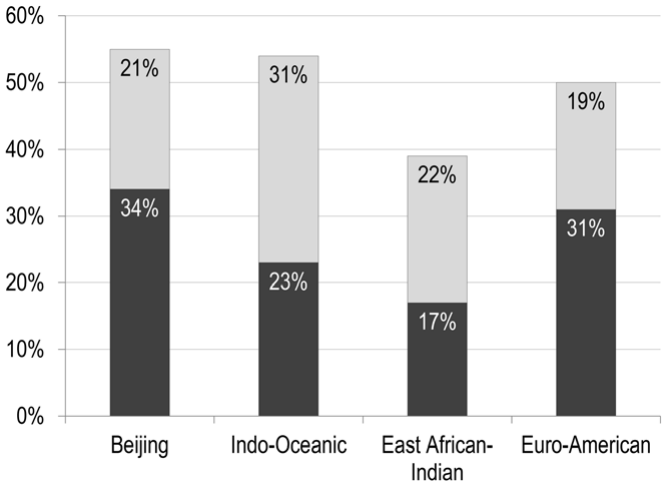
Estimated proportion of recent versus remote tuberculosis infection among derivation cohort contacts. A comparison between close contacts of active pulmonary TB patients within each MTB lineage group. Light coloured bars: proportion estimated to have remote TB infection based on the calculated probability of latent TB at the time of immigration to Canada (see methods for details). Dark coloured bars: remaining TST positive (out of total) assumed to have recent TB infection (i.e. equivalent to TST conversion). Abbreviations: TB = tuberculosis.

In this derivation cohort, as seen in Figure 1, the EAI lineage was less likely to be associated with RFLP clustered cases (0% vs. 19%, p<0.001) when compared to the other lineages. When we stratified our subjects based on the disease site (pulmonary and extra-pulmonary), the result among subjects with pulmonary TB only was the same (0 vs. 19%; p: 0.02). Close contacts of pulmonary TB patients with disease due to the EAI lineage were less likely to have positive TST (overall) (39% vs. 51%, p: 0.046). Using the methods described above to identify recent TB infections, the estimated proportions with recent TB infection (equivalent to TST conversion) among contacts of EAI and non-EAI infected patients were 17% and 29%, respectively (Figure 2). The EAI lineage was also less likely to be associated with instances of severe TB (0% vs. 10%, p: 0.025), whilst the proportion with drug resistant TB was identical (10% vs. 10%, p: 1.0), (Figure 1). Although not statistically significant, the Beijing lineage was associated with a somewhat higher proportion of positive TST among contacts (55% vs. 50%, p: 0.37) and drug resistance to one or more anti-TB drugs (14% vs. 10%, p: 0.22) as compared to the other lineages combined. These strains were also associated with a lower proportion in RFLP clusters (9% vs. 19%, p: 0.05) and severe disease (8% vs. 10%, p: 0.6). There were also no significant differences in primary outcomes, when comparing patients across the other MTB lineages.

Considering the other clinical features (secondary outcomes), the EAI lineage was associated with a higher proportion of TB lymphadenitis (34% vs. 22%, p: 0.067) and extra-pulmonary TB (45% vs. 30%, p: 0.05), and lower proportion of weight loss (23% vs. 38%, p: 0.066) as compared to the other lineages; for more details about clinical features associated with each of the four MTB lineages see Table S2.

### Validation Cohort

In total, 91 patients with active TB, extractable DNA, and adequate clinical information were identified, along with 229 close contacts of the pulmonary TB patients within this group; refer to Figure S2 for more details. Of those with active TB, 36 (40%) were infected with EAI lineage strains. The environmental and host-related risk factors were comparable between the EAI and non-EAI groups, except for the TB prevalence in the patients' countries of origin (Table 1). For this validation cohort, the EAI lineage was again associated with a significantly lower proportion of positive TST among contacts (44% vs. 58%, p: 0.04), and a lower proportion with estimated recent infection (17% versus 33%, p: 0.01). The EAI lineage was also associated with somewhat fewer RFLP clustered cases (3% vs. 13%, p: 0.14) when compared to the other lineages. Because of the small number of patients with severe TB, 4 among the EAI group (2 of whom died during TB therapy due to myocardial infarction and brain malignancy) and 3 among the non-EAI group, we could not assess any associations between strain lineage and clinical severity in this second cohort.

### Multivariate analysis

After adjustment for age and probability of previous latent TB, the EAI lineage was associated with significantly lower odds of having a positive TST among contacts in the derivation cohort (adjusted OR 0.6: [95% confidence interval 0.4–0.9]) and the validation cohort (0.5: [0.3–0.9]). The odds of being part of an RFLP cluster or having severe TB, were also significantly lower among the EAI group in the derivation cohort (adjusted ORs = 0.3: [<0.001–0.6] and 0.3: [<0.001–0.8] respectively), but were not significantly different in the validation cohort (adjusted ORs = 0.5: [0.1–1.8] and 1.4: [0.4–11] respectively); see Tables 2 and 3 for details.

**Table 2:** Primary outcomes associated with East African-Indian lineage and other lineages combined in the derivation cohort.

	East African-Indian (EAI)	Non-EAI*	P-value
+TST			
N/Tot. (%)	29/74 (39)	647/1265 (51)	0.046
Crude OR (CI)	0.6 (0.4–0.99)	1.0 (Ref.)	
Adjusted OR (CI)†	0.6 (0.4–0.93)	1.0 (Ref.)	
RFLP Cluster			
N/Tot. (%)	0/41 (0)	124/637 (19)	<0.001
Crude OR (CI)‡	0.3 (<0.001–0.6)	1.0 (Ref.)	
Adjusted OR (CI)§	0.3 (<0.001–0.6)	1.0 (Ref.)	
Severe TB^ll^			
N/Tot. (%)	0/41 (0)	64/637 (10)	0.025
Crude OR (CI)‡	0. 4 (<0.001–0.94)	1.0 (Ref.)	
Adjusted OR (CI)§	0.3 (<0.001–0.8)	1.0 (Ref.)	
Drug resistance			
N/Tot. (%)	4/41 (10)	62/622 (10)	0.965
Crude OR (CI)	1.0 (0.4–3.0)	1.0 (Ref.)	
Adjusted OR (CI)**	1.2 (0.4–3.4)	1.0 (Ref.)	

Abbreviations: N = number; Tot = total; +TST = positive tuberculin skin test; CI = 95% confidence interval; Ref. = reference; TB = tuberculosis.

*Indicates Beijing, Indo-Oceanic and Euro-American lineages combined;

† adjusted for age and probability of latent TB at the time of immigration, contact's sex and comorbidity information are not available;

‡ estimated using exact logistic regression analysis;

§ adjusted for age, adjustment for multiple covariates was technically difficult in the presence of zero events;

ll severe TB indicates disseminated TB, TB meningitis, extensive bilateral lung consolidation and/or death during the course of TB therapy;

**adjusted for age, sex, co-morbidities, smoking history, alcohol abuse, IV drug use, history of previous TB, and TB prevalence in the countries of origin.

**Table 3:** Primary outcomes associated with East African-Indian lineage and other lineages combined in the validation cohort.

	East African-Indian (EAI)	Non-EAI	P-value
+TST			
N/Tot. (%)	38/86 (44)	83/143(58)	0.042
Crude OR (CI)	0.6 (0.3–0.98)	1.0 (Ref.)	
Adjusted OR (CI)*	0.5 (0.3–0.89)	1.0 (Ref.)	
RFLP Cluster			
N/Tot. (%)	1/36 (2.8)	7/55 (12.7)	0.140
Crude OR (CI)†	0.5 (0.1–1.3)	1.0 (Ref.)	
Adjusted OR (CI)‡	0.5 (0.1–1.8)	1.0 (Ref.)	
Severe TB§			
N/Tot. (%)	4/36 (11)	3/55 (5.4)	0.428
Crude OR (CI)†	1.5 (0.6–4)	1.0 (Ref.)	
Adjusted OR (CI)‡	1.4 (0.4–11)	1.0 (Ref.)	
Drug resistance			
N/Tot. (%)	7/35 (20)	6/53 (11)	0.261
Crude OR (CI)†	1.4 (0.7–2.8)	1.0 (Ref.)	
Adjusted OR (CI)‡	1.2 (0.6–2.8)	1.0 (Ref.)	

Abbreviations: N = number; Tot. = total; +TST = positive tuberculin skin test; CI = 95% confidence interval; Ref. = reference; TB = tuberculosis.

*Adjusted for age and probability of latent TB at the time of immigration, contact's sex and comorbidity information are not available;

† estimated using exact logistic regression analysis;

‡ adjusted for age, sex, co-morbidities, smoking history, history of previous TB, and TB prevalence in the countries of origin;

§ severe TB indicates disseminated TB, TB meningitis, extensive bilateral lung consolidation and/or death during the course of TB therapy.

## Discussion

In our Montreal-based study, 4 major MTB lineages were identified and the associated clinical features were compared. In contrast to studies carried out in other populations where Beijing isolates were associated with greater virulence, and transmissibility [11], [22], [23] or with drug–resistance [11], [24], these features were not associated with this lineage in Montreal. On the other hand, in our study the EAI lineage was less likely to cause infection or disease transmission, or to be associated with severe clinical manifestations when compared to the other 3 MTB lineages. Considering the geographic distribution of the EAI lineage, our finding is concordant with laboratory evidence from the 1960's suggesting that MTB isolates from Southern Indian patients were less virulent compared to isolates from British patients [25]. Although very difficult to confirm, it is possible that at least some of these Southern Indian isolates belonged to the EAI lineage.

In contrast to our findings, a strain belonging to the EAI lineage (the “CH strain”) has recently been identified as being responsible for a large tuberculosis outbreak in the United Kingdom [26]. Despite the identification of some anti-inflammatory characteristics of the CH-MTB strain that can influence the innate immune response, and which makes it plausible for this strain to be highly transmissible, no population based epidemiological study has compared the transmissibility of this strain to other MTB strains [27]. It is also interesting to note that the genomic deletion that was identified as being responsible for the anti-inflammatory properties of the CH bacteria (the RD750 deletion) is also absent from each of our EAI strains.

Our study limitations include the relatively small number of patients infected with the EAI lineage, which is the group of interest in this study. In addition, there are potential confounding effects related to patient's ethnicity and socioeconomic status, which were difficult to control in our analysis. Studying TB related outcomes in a country like Canada, where the TB control program has substantial resources, can substantially limit TB transmission and reduce unfavourable disease outcomes, limiting our power to detect important relationships. *M. africanum*, which has been associated with a lower rate of disease transmission compared to other MTB strains [16], was not included in our study because of the very small numbers of patients infected with these strains. Considering that disease transmission is less likely from patients with extra-pulmonary TB, transmissibility of EAI lineage as compared to other MTB lineages might have been less because more patients with EAI disease had extra-pulmonary TB in our derivation cohort. However, when our analysis was restricted to contacts of patients with pulmonary TB, or to RFLP clustering among pulmonary TB cases, TB transmissibility was reduced among EAI-MTB infected group.

A major strength of our study was that we employed a second validation cohort to test for potentially spurious associations in the first, derivation cohort. By focusing on a specific sub-population with a common geographic background, where the EAI lineage is most frequently isolated, we were able to show that the lower risk of TB transmission among EAI-infected patients and their contacts was reproducible across different time-windows in Montreal, and within a more restricted comparison group – in an attempt to limit the ethnic (and presumed genetic) diversity of the TB cases and their contacts. Although the proportions of RFLP clustered cases appear different in the two cohorts (among EAI groups were 0% [95%CI: 0–9%] versus 3% [0.1–15%] and among the non-EAI groups were 19% [17–23%] versus 13% [6–23%]), we think that this is mainly related to random variations between the two samples, since the estimates from the two samples are similar and their 95% CIs are overlapping. Another strength is related to the global distribution of our study sample; 83% of patients were foreign-born from 80 different countries in 16 different geographic regions [3]. Because of the phylogeographic diversity of the major MTB lineages, such a global study sample is crucial to perform comparative analyses to identify associations between individual strain lineages (or sub-lineages thereof) and clinical disease features [3], [5].

Our study observations, which characterize major MTB lineages based on a comprehensive clinical assessment of a diverse population, provides some interesting insights into the potential pathogenicity of the EAI-MTB lineage and suggests some potential clinical implications, although independent validation of these findings in another setting is needed. Therefore we suggest studies using deletion analysis to classify strains in the Indian sub-continent area to see whether the observed associations in this study are also seen in the country of origin of these bacteria. As part of future risk assessments, MTB lineage detection could influence the management plan among TB patients, or the extent of TB screening among contacts of a TB patient.

### Conclusions

This study provides evidence that the East African-Indian lineage strains are associated with a lower risk of transmission and, possibly, a lower risk of developing severe forms of active disease. In addition, for our Montreal cohort, Beijing lineage strains were found not to be associated with enhanced transmissibility or disease severity.

## Supporting Information

Figure S1 **Population selection in the derivation cohort.** (A) Cases. (B) Contacts. Abbreviations: PTB = pulmonary tuberculosis.
(TIF)

Figure S2 **Population selection in the validation cohort.** (A) Cases. (B) Contacts. Abbreviations: PTB = pulmonary tuberculosis.
(TIF)

Table S1 **Patient characteristics associated with East African-Indian and other** ***Mycobacterium tuberculosis*** **lineages in the derivation cohort.** Abbreviations: N = number; TB = tuberculosis; IV = intravenous. * P-value for difference between all 4 groups, using chi square or analysis of variance (see methods; degrees of freedom = 3); ^†^estimated TB prevalence >150 per 100, 000, based on the 2009 World Health Organization reports (1); ^‡^ measuring p-value is not appropriate because of pre-assigned differences; **^§^** number of years of stay in Canada since immigration – for foreign born only; ^ll^ co-morbidities include chronic pulmonary diseases, diabetes, cardiac diseases, renal diseases, liver diseases, and non-HIV immunosuppressive disorders (e.g. malignancies, immunosuppressive medications); ** 73% missing values.
(DOC)

Table S2 **Clinical features associated with major** ***Mycobacterium tuberculosis*** **lineages in derivation cohort.** Abbreviations: N = number; TB = tuberculosis. *Includes all non-East African-Indian lineages combined; ^†^ p-value for difference between East African-Indian lineage and other lineages combined, using chi square (see methods; degrees of freedom = 1); ^‡^the disease site is missing for 3 patients; ^§^includes patients who have both pulmonary and extra-pulmonary combined.
(DOC)
