# Supplementary figures and images for: Reduced Transmissibility of East African Indian Strains of Mycobacterium tuberculosis
Source: PLoS One. 2011 Sep 19;6(9):e25075. doi: 10.1371/journal.pone.0025075 (PMC3176299; doi:10.1371/journal.pone.0025075)

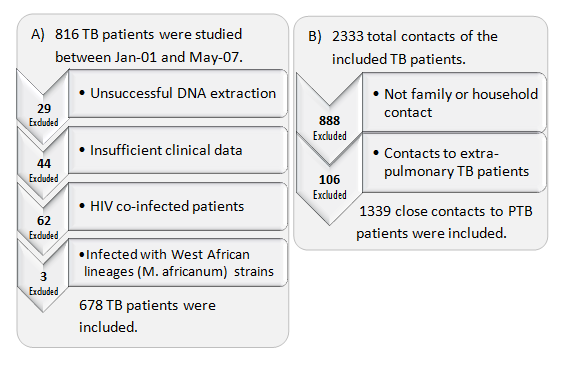

Supplement: Figure S1 — Population selection in the derivation cohort. (A) Cases. (B) Contacts. Abbreviations: PTB = pulmonary tuberculosis. (TIF) [file pone.0025075.s001.tif]

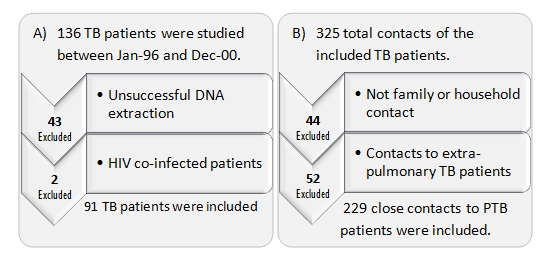

Supplement: Figure S2 — Population selection in the validation cohort. (A) Cases. (B) Contacts. Abbreviations: PTB = pulmonary tuberculosis. (TIF) [file pone.0025075.s002.tif]
