# Supplementary material for: Reduced Transmissibility of East African Indian Strains of Mycobacterium tuberculosis
Source: PLoS One. 2011 Sep 19;6(9):e25075. doi: 10.1371/journal.pone.0025075 (PMC3176299; doi:10.1371/journal.pone.0025075)
Supplement: Table S1 — Patient characteristics associated with East African-Indian and other Mycobacterium tuberculosis lineages in the derivation cohort. Abbreviations: N = number; TB = tuberculosis; IV = intravenous. * P-value for difference between all 4 groups, using chi square or analysis of variance (see methods; degrees of freedom = 3); †estimated TB prevalence >150 per 100, 000, based on the 2009 World Health Organization reports (1); ‡ measuring p-value is not appropriate because of pre-assigned differences; § number of years of stay in Canada since immigration – for foreign born only; ll co-morbidities include chronic pulmonary diseases, diabetes, cardiac diseases, renal diseases, liver diseases, and non-HIV immunosuppressive disorders (e.g. malignancies, immunosuppressive medications); ** 73% missing values. (DOC) [file pone.0025075.s003.doc]

**TABLE S1. Patient characteristics associated with East African-Indian and other *Mycobacterium tuberculosis* lineages in the derivation cohort.**

Abbreviations: N = number; TB = tuberculosis; IV = intravenous.
* P-value for difference between all 4 groups, using chi square or analysis of variance (see methods; degrees of freedom = 3); ^†^estimated TB prevalence > 150 per 100, 000, based on the 2009 World Health Organization reports (1); ^‡^ measuring p-value is not appropriate because of pre-assigned differences; **^§^** number of years of stay in Canada since immigration – for foreign born only; ^ll^ co-morbidities include chronic pulmonary diseases, diabetes, cardiac diseases, renal diseases, liver diseases, and non-HIV immunosuppressive disorders (e.g. malignancies, immunosuppressive medications); ** 73% missing values.

|  | **Beijing** | | **Indo-Oceanic** | **East African-Indian** | **Euro-American** | **P-value*** |
| --- | --- | --- | --- | --- | --- | --- |
|  | | | | | | |
| 1. **Risk factors –TB patients (i.e. index cases); total number = 678.** | | | | | | |
| **Total number** | **65** | | **120** | **41** | **452** |  |
| Age, mean (SD) | 44 (21) | | 45 (18) | 47 (21) | 44 (21) | 0.83 |
| Male sex, N (%) | 29 (45) | | 66 (55) | 20 (49) | 242 (55) | 0.42 |
| Immigrants from high TB prevalence country**^†^**, N (%) | 50 (81) | | 106 (90) | 36 (88) | 220 (50) | **‡** |
| Years in Canada**^§^**, mean(SD) | 11 (13) | | 10 (9) | 8 (10) | 10 (13) | 0.56 |
| Co-morbidities^ll^, N (%) | 20 (31) | | 45 (38) | 13 (32) | 150 (33) | 0.76 |
| History of smoking, N (%) | 10 (20) | | 28 (29) | 5 (19) | 117 (32) | 0.17 |
| Alcohol abuse, N (%) | 8 (16) | | 11 (12) | 4(15) | 70 (20) | 0.32 |
| IV drug use, N (%) | 0 | | 0 | 0 | 4 (1) | 0.78 |
| Past TB infection, N (%) | 6 (9) | | 12 (10) | 2 (5) | 51 (11) | 0.61 |
| 1. **Risk factors – close contacts of pulmonary TB patients; total number = 1339.** | | | | | | |
| **Total number** | | **110** | **279** | **74** | **876** |  |
| Age, mean (SD) | | 32 (22) | 29 (20) | 35 (23) | 29 (20) | 0.15 |
| Male sex^**^, N (%) | | 24 (44) | 48 (58) | 13 (45) | 98 (49) | 0.38 |
| Immigrants from high TB prevalence country**^†^**, N (%) | | 74 (67) | 201(72) | 46 (62) | 400 (46) | **‡** |
| Years in Canada**^§^**, mean (SD) | | 11 (7) | 9 (9) | 10 (10) | 9 (11) | 0.62 |
