# Supplementary material for: Reduced Transmissibility of East African Indian Strains of Mycobacterium tuberculosis
Source: PLoS One. 2011 Sep 19;6(9):e25075. doi: 10.1371/journal.pone.0025075 (PMC3176299; doi:10.1371/journal.pone.0025075)
Supplement: Table S2 — Clinical features associated with major Mycobacterium tuberculosis lineages in derivation cohort. Abbreviations: N = number; TB = tuberculosis. *Includes all non-East African-Indian lineages combined; † p-value for difference between East African-Indian lineage and other lineages combined, using chi square (see methods; degrees of freedom = 1); ‡the disease site is missing for 3 patients; §includes patients who have both pulmonary and extra-pulmonary combined. (DOC) [file pone.0025075.s004.doc]

|  | **East African-Indian** | | **Non-East African-Indian** | | | | | **P-value^†^** |
| --- | --- | --- | --- | --- | --- | --- | --- | --- |
|  |  |  | Indo-Oceanic | Beijing | Euro-American | Combined* | |  |
|  | | N (%) | N (%) | N (%) | N (%) | N (%) | |  |
| 1. **Disease site**^‡^ | |  |  |  |  |  | |  |
| Pulmonary | | 18 (45) | 70 (59) | 39 (60) | 294 (65) | 403 (63) | | 0.02 |
| Extra-pulmonary | | 18 (45) | 41 (34) | 22 (34) | 128 (28) | 191 (30) | | 0.05 |
| Both | | 4 (10) | 8 (7) | 4 (6) | 29 (6) | 41 (6) | | 0.38 |
| 1. **Clinical characteristics** **– all TB patients** | | | | | | | | |
| Total number | | 41 | 120 | 65 | 452 | 637 |  | |
| TB lymphadenitis | | 14 (34) | 29 (24) | 16 (25) | 94 (21) | 139 (22) | 0.07 | |
| Fever | | 14 (40) | 42 (36) | 22 (38) | 148 (35) | 212 (36) | 0.60 | |
| Night sweats | | 7 (20) | 17 (15) | 11 (19) | 90 (22) | 118 (20) | 0.97 | |
| Fatigue | | 5 (15) | 17 (16) | 12 (21) | 84 (21) | 113 (20) | 0.45 | |
| Weight loss | | 8 (23) | 47 (41) | 20 (33) | 162 (38) | 229 (38) | 0.07 | |
| Loss of appetite | | 8 (23) | 23 (21) | 12 (21) | 95 (23) | 130 (23) | 0.98 | |
| 1. **Clinical characteristics – patients with pulmonary disease**^§^ | | | | | | | | |
| Total number | | 22 | 78 | 43 | 323 | 444 |  | |
| Cough | | 16 (70) | 53 (69) | 29 (69) | 221 (71) | 303 (70) | 0.94 | |
| Sputum | | 6 (29) | 33 (44) | 22 (56) | 156 (51) | 211 (50) | 0.06 | |
| Hemoptysis | | 3 (14) | 12 (17) | 9 (25) | 49 (16) | 70 (17) | 1.00 | |
| AFP smear positive | | 9 (39) | 39 (49) | 21 (49) | 166 (52) | 226 (51) | 0.26 | |
| Cavitary lesion | | 7 (30) | 22 (28) | 10 (23) | 95 (29) | 127 (28) | 0.84 | |

Abbreviations: N = number; TB = tuberculosis.
*Includes all non-East African-Indian lineages combined; ^†^ p-value for difference between East African-Indian lineage and other lineages combined, using chi square (see methods; degrees of freedom = 1); ^‡^the disease site is missing for 3 patients; ^§^includes patients who have both pulmonary and extra-pulmonary combined.

**TABLE S2. Clinical features associated with major *Mycobacterium tuberculosis* lineages in derivation cohort.**
